# *Caffeic acid phenethyl ester* derivative exerts remarkable anti-hepatocellular carcinoma effect, non-inferior to sorafenib, in vivo analysis

**DOI:** 10.1038/s41598-024-65496-1

**Published:** 2024-06-24

**Authors:** Lei Gong, Wenzhen Wang, Fei Yu, Zenghua Deng, Nan Luo, Xinjing Zhang, Jianfen Chen, Jirun Peng

**Affiliations:** 1 Department of Surgery, Beijing Shijitan Hospital, Capital Medical University, Beijing, 100038 People’s Republic of China; 2 Department of Urology, Second Affiliated Hospital, Shandong University, Jinan, 250021 People’s Republic of China; 3 Center of Hepatopancreatobiliary Diseases, Beijing Tsinghua Changgung Hospital, School of Clinical Medicine, Tsinghua University, Beijing, 102218 People’s Republic of China

**Keywords:** Hepatocellular carcinoma, *Caffeic acid phenethyl ester* derivative, Sorafenib, Non-inferiority, In vivo analysis, Drug development, Molecular medicine

## Abstract

*Caffeic acid phenethyl* ester (CAPE) and its derivatives exhibit considerable effects against hepatocellular carcinoma (HCC), with unquestioned safety. Here we investigated CAPE derivative 1ʹ (CAPE 1ʹ) monotherapy to HCC, compared with sorafenib. HCC Bel-7402 cells were treated with CAPE 1ʹ, the IC50 was detected using CCK-8 analysis, and acute toxicity testing (5 g/kg) was performed to evaluate safety. In vivo, tumor growth after CAPE 1ʹ treatment was evaluated using an subcutaneous tumor xenograft model. Five groups were examined, with group 1 given vehicle solution, groups 2, 3, and 4 given CAPE 1ʹ (20, 50, and 100 mg/kg/day, respectively), and group 5 given sorafenib (30 mg/kg/day). Tumor volume growth and tumor volume-to-weight ratio were calculated and statistically analyzed. An estimated IC50 was 5.6 µM. Acute toxicity tests revealed no animal death or visible adverse effects with dosage up to 5 g/kg. Compared to negative controls, CAPE 1ʹ treatment led to significantly slower increases of tumor volume and tumor volume-to-weight. CAPE 1ʹ and sorafenib exerted similar inhibitory effects on HCC tumors. CAPE 1ʹ was non-inferior to sorafenib for HCC treatment, both in vitro and in vivo. It has great potential as a promising drug for HCC, based on effectiveness and safety profile.

## Introduction

Hepatocellular carcinoma (HCC) is among the most common malignant tumors, causing substantial numbers of tumor-related deaths worldwide^1,2^. Surgery is the principal method of obtaining a radical cure, including hepatectomy and liver transplantation^3,4^. However, most HCC patients are diagnosed when in middle or late disease stages, which are characterized by a large tumor burden, vessel invasion, extra-hepatic metastasis, and poor liver function, etc^5^. Therefore, most patients no longer have the opportunity for radical treatment. For these patients, systemic drug therapy plays an important role in treatment. Drug treatment can control disease progression, and prolong survival over time, especially in the present era of targeted therapy and immunotherapy^6–8^.

Systemic drug therapy includes molecular targeted drug therapy, immunotherapy, and chemotherapy, as well as drugs for the treatment of basic liver diseases, such as anti-viral treatment, liver protective treatment, and supportive treatment^9–11^. The options for drug have been rapidly advancing. Even for surgery candidates, drugs can reduce recurrence, prolong the recurrence-free survival period after surgery, and improve patients’ quality of life^12,13^.

Sorafenib is the first targeted drug for treatment of advanced HCC^14^. It is a multi-kinase inhibitor that can inhibit tumor cell proliferation, promote tumor cell apoptosis, and slow tumor angiogenesis^15,16^. Although sorafenib is effective, it has relatively serious adverse effects for some patients, and it is unfortunately easy for patients to develop sorafenib-resistance at an early stage^17–19^. Thus, there remains a need for new compounds having similar or better effectiveness but lower toxicity.

Natural products have great potential for application due to their low toxicity or non-toxicity and significant therapeutic effects. Many studies have supported this claim^20–22^. Among them, *Caffeic acid phenethyl ester* (CAPE), a phenolic compound originally derived from natural propolis, has been reported to exert notable anticancer activity^23,24^. It can inhibit tumor proliferation and metastasis (including HCC), and also somehow protects organ function^25–27^. Despite these benefits, the clinical therapeutic application of CAPE is limited due to its poor absorption and rapid metabolism. These limitations may be overcome by structural modification through the synthesis of CAPE-based derivatives.

A new compound was synthesized with the aid of the School of Pharmacy of Peking University. The derivative named CAPE 1ʹ overcomes the disadvantages of CAPE, while enhancing its overall efficacy and hindering drug resistance. In this study, we report the anti-HCC activity of CAPE 1ʹ compared to sorafenib, the classic therapeutic drug for HCC.

## Materials and methods

### Materials

CAPE 1ʹ was synthesized by the School of Pharmacy of Peking University. The HCC cell line BEL-7402, initially obtained from the American Type Culture Collection, was cryopreserved in our own lab in liquid nitrogen. Sodium carboxymethylcellulose was purchased from Sigma-Aldrich (St. Louis, MO, USA), and sorafenib from Bayer Pharmaceutical Company in Germany. The animals used in this study were male specific pathogen-free BALB/C nude mice purchased from Vital River Corp., Beijing. Kunming mice were purchased from the Academy of Military Medical Sciences. All experiments were approved by the Laboratory Animal Ethics Committee of Peking University People’s Hospital (No. 20110312). Animals enrolled in the experiment were treated in accordance with the standards set forth in the 8th Edition of Guide for the Care and Use of Laboratory Animals. All of the mice were sacrificed via exsanguination following anesthesia with 5% isoflurane. The study is reported in accordance with ARRIVE guidelines.

### HCC cell inhibition analysis

HCC BEL-7402 cells were cultured in RPMI 1640 medium. Cell viability was determined by 0.4% trypan blue staining (viewed by inverted microscope), and propidium iodide (PI) staining (analyzed by flow cytometry). Viable cells were cultured at a density of 5 × 10^3^/well in 96-well plates for 24 h. CAPE 1ʹ was dissolved in DMSO and diluted into various concentrations using 5‰ sodium carboxymethyl cellulose solution.

Six wells of HCC cells were treated with CAPE 1ʹ at concentrations of 0, 2.5, 5, 10, 20, 40, 80, and 160 µM. The culture medium volume was adjusted to 200 µL with RPMI1640, and then the HCC cells were cultured for 48 h. The CCK-8 method (Tongren Chemical Research Institute, Japan) was used to test the cell survival rate after treatment. The optical density of each well was determined at a wavelength of 450 nm. The 50% inhibitory concentration (IC50) was calculated for the HCC BEL-7402 cells.

### Animal acute toxicity test

A total of 40 Kunming mice (half male and half female, 4 weeks old, weighing about 20 g) were randomly divided into an experimental group and control group, each containing 20 mice. Each mouse in the experimental group was administered the drug by gavage on the first day, while the control group was given solvent. The dose of 5 g/kg was established based on the pilot study, which used a dose of 2 g/kg. The mice were observed for toxic reactions and death, and the median lethal dose (LD50) was determined with reference to the literature^28,29^. After 14 days, the mice were sacrificed, and specimens of main organs (including the heart, liver, kidney, and lung) were collected to test for toxicity. The experiment was conducted following the guidelines for acute toxicity experiments of chemical drugs from the China Food and Drug Administration^30^.

### Subcutaneous tumor model establishment

Viable HCC BEL-7402 cells were subcutaneously implanted in male 4-week-old nude mice. Four weeks later, when tumors had formed, the established tumors were seeded from the tumor-burdened mice. These tumors served as the original specimens, and were cut into pieces of 2-mm diameter. These tumor pieces were then subcutaneously implanted into the backs of nude mice. After 2 weeks, when the tumors had grown, these implanted nude mice were prepared for the subsequent experiment.

### In vivo drug treatment

A total of 90 nude mice with **s**ubcutaneous tumors were randomly divided into an experimental group and control group, based on the ratio of transplanted tumor to body weight. These mice were then divided into five groups, with each group including 18 mice. One group was administered only the vehicle solution, as a negative control (group 1). Three groups were administered CAPE 1ʹ at different concentrations: 20 mg/kg/day (group 2), 50 mg/kg/day (group 3), and 100 mg/kg/day (group 4). The last group was given sorafenib (30 mg/kg/day) as a positive control (group 5).

Treatment was administered six days a week (skipping Sundays) for four weeks. Each day, we recorded the tumor size, body weight, adverse reactions, and other parameters. We calculated and analyzed the tumor volume (v = a * b^2^/2, where a is the long diameter in mm and b is the short diameter in mm), and the ratio of tumor volume to weight (ratio = v/w, where v is the tumor volume in mm^3^, and w is the body weight in g).

### Statistics

Statistical analyses were performed using SPSS Statistics 20.0 software. Data are presented as mean ± standard deviation. Student's t-test test was used for comparisons between two groups (group 1 vs group 2, 3, 4, 5, respectively) of samples, and Tukey’s test in one-way ANOVA was used for comparisons between multiple groups (All 5 groups) of samples. The Dunnett statistical test was used to compare each of the treatments (different concentration of the compound) with a single control. *P* < 0.05 was considered statistically significant.

### Ethical approval

All experiments were approved by the Laboratory Animal Ethics Committee of Peking University People’s Hospital.

## Results

### IC50 analysis

**Figure 1:**
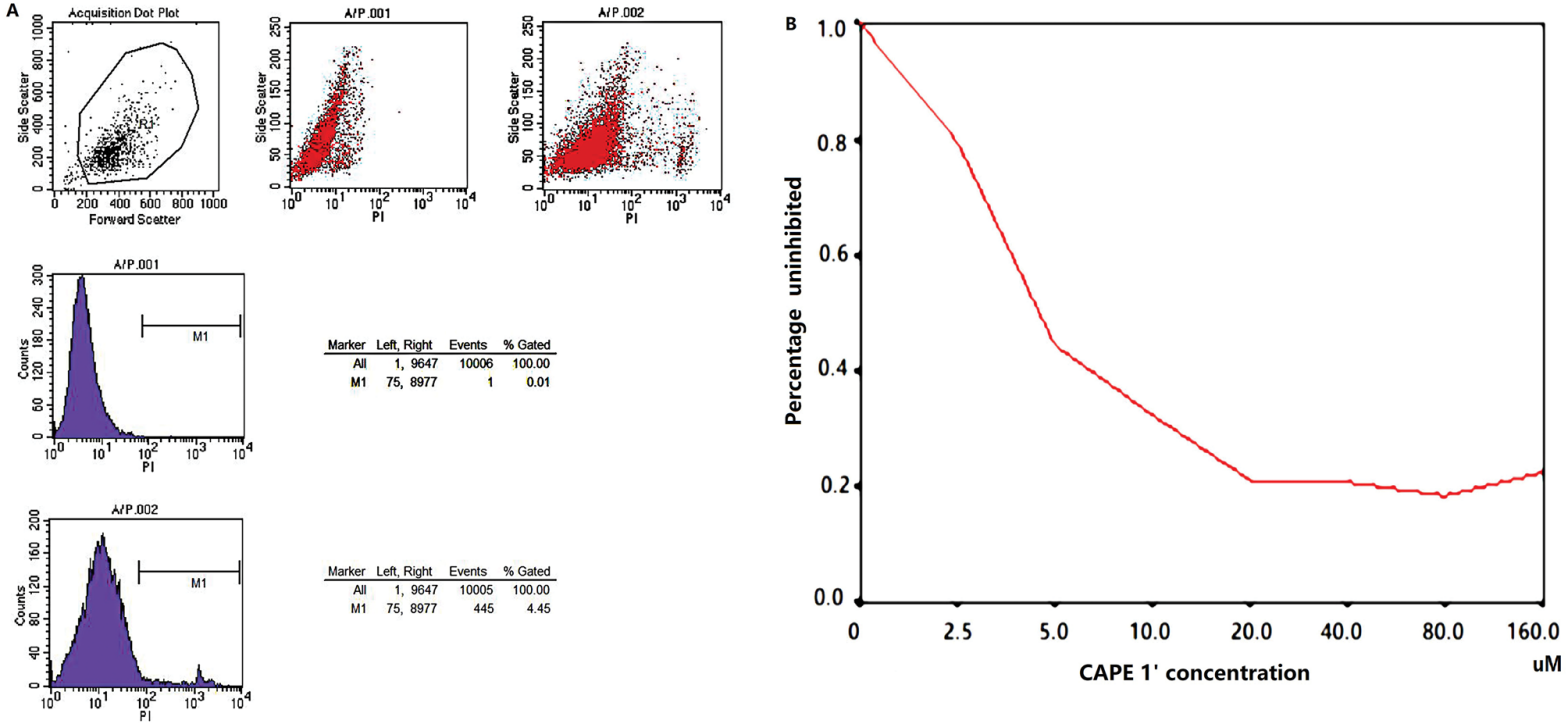
(**A**) Flow cytometry showing cell viability of about 95%. (**B**) Inhibition curve, showing that the effect is enhanced with increasing dosage.

HCC BEL-7402 cells were cultured and seeded as planned. Cell viability was estimated to be over 90% by 0.4% trypan blue staining and above 95% based on PI staining (Fig. 1A). Several CAPE derivatives were tested by in vitro analysis. CAPE 1ʹ was the most effective derivative, with the lowest IC50 (5.6 µM). Figure 1B presents the inhibition curve. At the higher tested concentrations, about 80% of cells were suppressed.

### Acute toxicity test

**Figure 2:**
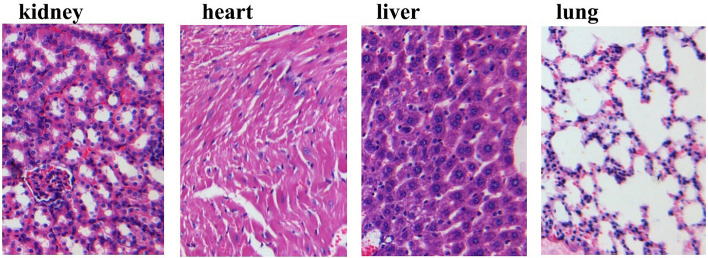
CAPE 1ʹ exhibited no damage to vital organs such as the kidney, heart, liver and lung.

We conducted a preliminary experiment in which four mice were administered a dose of 2 g/kg by gavage. No mice died during 7 days of observation. Therefore, according to the Guiding Principles for Acute Toxicity Experiment of Chemical Drugs, we proceeded to conduct the formal experiment with a dose of 5 g/kg. One mouse in the experimental group died on the second day, and was found to have intrathoracic perfusion fluid after dissection. This death was considered to be caused by improper intragastric perfusion on the first day. The other mice showed a 100% survival rate, and no toxic reaction was observed. All the data was summarized in Table 1. Since the drug dosage used in this experiment was up to 5 g/kg, and no animal death or visible side effects were observed, the LD50 cannot be calculated. CAPE-1′ can be considered a non-toxic or low-toxic drug, without risk of serious acute poisoning to vital organs (Fig. 2).

**Table 1:** Results of toxic reaction.

Group	Number	Weight (g)			Dose (g/kg)	Toxic reaction	Death
		Day 1	Day 14	Increase			
Experiment	19	26.09 ± 1.97	37.22 ± 6.10	11.13 ± 5.01	5	No	0
Control	20	24.73 ± 1.46	35.93 ± 5.42	11.20 ± 5.38	0	No	0

### In vivo treatment

After the mice were divided into groups, their baseline characteristics were analyzed. Single-factor analysis of variance revealed a *P* value of 0.973 for baseline tumor volume, and a *P* value of 0.934 for the baseline ratio of tumor volume to weight. This indicates that the groups did not significantly differ in tumor volume or ratio of tumor volume to weight at baseline.

At end of the experiment, each group's survivorship after the four weeks was 15/18, 16/18, 15/18, 15/18, and 14/18, respectively. We calculated the tumor volume growth and ratio of tumor volume to weight in the mice of each group. To assess the anti-tumor effect reflected by tumor growth, these two parameters were compared between groups (negative control vs all experiment groups respectively). For tumor volume growth, the *P* values were as follows: *P*_(1,2)_ = 0.014, *P*_(1,3)_ = 0.042,* P*_(1,4)_ = 0.313, and* P*_(1,5)_ = 0.022. This analysis revealed that each experimental group exhibited significantly slower tumor volume growth compared to the negative control group. For the ratio of tumor volume to weight, the *P* values were as follows: *P*_(1,2)_ = 0.004, *P*_(1,3)_ = 0.007, and *P*_(1,4)_ = 0.105, *P*_(1,5)_ = 0.008.

**Figure 3:**
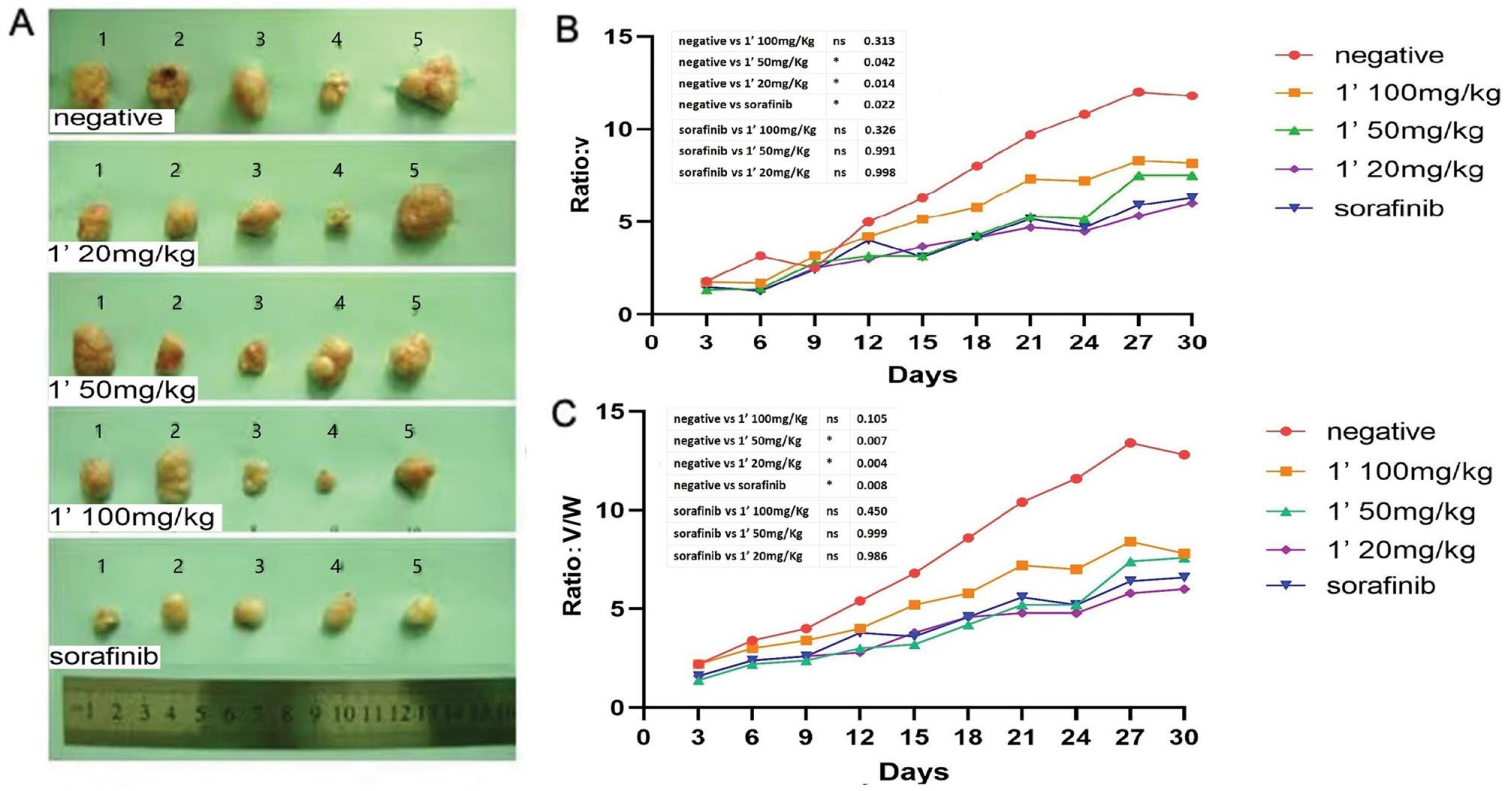
(**A**) Tumor specimen from each group. (**B**, **C**) Inhibitory curves for each group showing the tumor volume increase (**B**), and ratio of tumor volume to weight (**C**). ns, No significance, * *P* < 0.05, ** *P* < 0.01 compared with control group.

**Figure 4:**
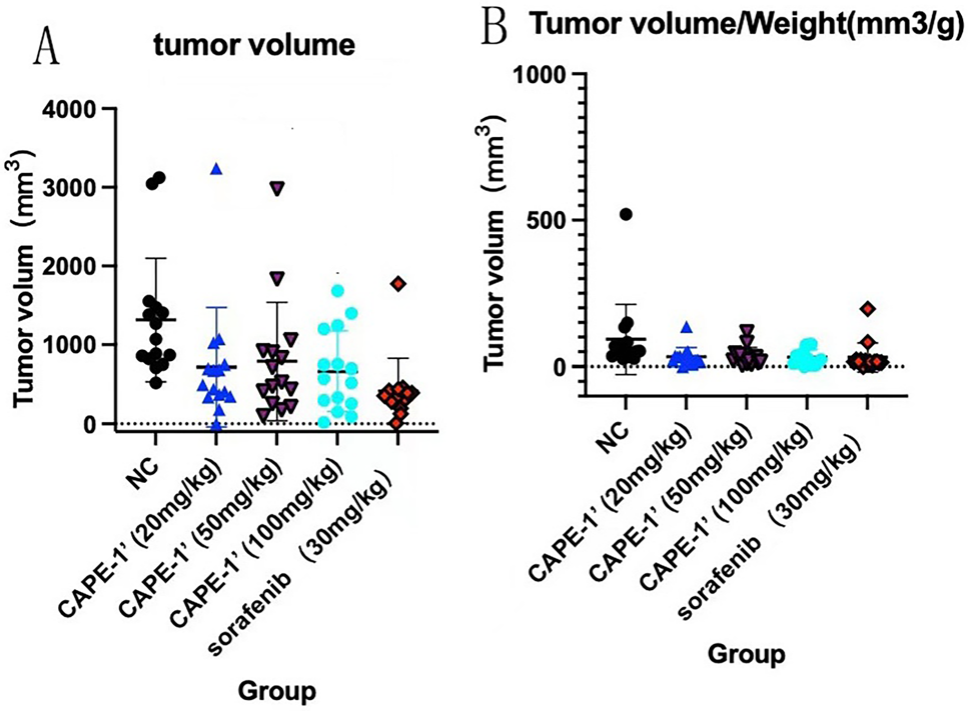
Scattergrams for each group, showing the tumor volume increase (**A**), and ratio of tumor volume to weight (**B**).

Compared to sorafenib treatment, CAPE 1ʹ treatment had a remarkable inhibitory effect on the HCC tumor—with the CAPE 1ʹ groups and the sorafenib group showing similar anti-HCC effects. For tumor volume growth, the *P* values were as follows: *P*_(2,5)_ = 0.998, *P*_(3,5)_ = 0991, and *P*_(4,5)_ = 0.326. For the ratio of tumor volume to weight, the *P* values were as follows: *P*_(2,5)_ = 0.986, *P*_(3,5)_ = 0.999, and *P*_(4,5)_ = 0.450. Even at the lower dosage of 20 mg/kg, CAPE 1ʹ had an obvious inhibitory effect on HCC, non-inferior to sorafenib. These findings are displayed in Figs. 3, 4.

## Discussion

The results of the present study demonstrated that CAPE 1ʹ was one of most effective inhibitory agents against HCC, based on both in vitro and in vivo analysis. In fact, its effects were almost non-inferior to those of the traditional drug sorafenib. Notably, CAPE 1ʹ exhibited no obvious toxicity, making it potentially one of most promising therapeutic drugs for HCC.

There are plenty of research prospects for natural products ^31–33^. Notably, CAPE is a natural compound derived from propolis, which shows various biological activities—with the most important and promising being its anti-tumor properties^34,35^. To overcome the physical and chemical inertness of CAPE, and to enhance its anti-tumor effect, a series of derivatives were synthesized and tested. Many experiments have been performed to test these derivatives’ effects on a variety of tumors^36,37^. Initial experiments show that these agents have considerable anti-tumor activity, but there remains a lack of in vivo experiments, especially clinical investigations.

CAPE derivatives have shown considerable anti-tumor activity in liver cancer. Liu et al. ^38^. designed a series of derivatives, authenticated their antineoplastic activity in vitro, and calculated their IC50. At same time, preliminary animal experiments were conducted. Compared with these previous compounds, our presently tested compound (CAPE 1ʹ) had a lower IC50 and substantially better effect, emphasizing the importance of in vivo analysis. However, all of these CAPE derivatives are mostly synthesized in the laboratory, with low yields^37,39,40^. They generally cannot be obtained commercially, which limits the ability to perform relevant experimental comparisons. It is also difficult to produce uniform derivatives. Future studies are needed to examine these issues.

The anti-tumor effects of CAPE have mainly been demonstrated in animal experiments. No previous study has performed a non-inferior effect comparison between CAPE and the most commonly used drugs in clinical settings. For advanced liver cancer, sorafenib is a standard first-line treatment method that has been widely used in clinical practice for almost two decades^41^. Thus, here we compared our derivative CAPE 1′ with sorafenib, and found that they showed comparable efficacy under the same experimental conditions. Although most often tolerable, sorafenib can have some severe adverse effects^42,43^. Importantly, CAPE 1' showed much better safety, with seemingly no obvious toxicity observed.

Investigations on CAPE's anti-tumor activity demonstrate that it produces cell cycle arrest, limits growth and transcription factor expression, and inhibits invasion, metastasis, and angiogenesis. It can also relieve the negative effects of anti-cancer medications, prevent and reverse the carcinogenic effects of some viruses and chemicals, and boost tumor cell apoptosis^44^. Many signal transduction pathways, including PI3K/Akt, p38MAPK, JNK, and non-classical Wnt pathways, are closely associated with its molecular mechanism^45,46^. For CAPE 1’, the mechanism may be somehow similar. It should, nevertheless, be confirmed in the next lab communications. Therefore, more research to clarify the molecular processes will be organized.

Many studies show that CAPE has cytotoxicity towards a variety of tumor cells and does not harm normal cells^27,47^. It also somehow protects organ function. Our present study demonstrated that the derivative CAPE 1ʹ showed significant time- and dose-dependent effects against HCC, suggesting great potential for HCC treatment. Increasing the dose of CAPE 1ʹ seemed to yield much better tumor suppression. Considering its good safety profile and antineoplastic effect, application in clinical trials is very realistic. In the present era of comprehensive therapy for HCC, monotherapy is not preferred^48,49^. CAPE, especially its improved derivative CAPE 1ʹ, can be used for auxiliary application in combination with other clinically approved drugs, such as sorafenib. In this regimen, CAPE 1ʹ could provide organ protection along with anti-tumor effects. A collaborative clinical trial will be carried out in the future following the elucidation of a more definitive molecular mechanism.

## Conclusion

In conclusion, the derivative CAPE 1ʹ exhibited considerable anti-HCC effect, comparable to that of sorafenib. The anti-tumor effect was improved by using an increased dosage. Importantly, in vivo animal experiments also revealed that CAPE 1ʹ had a favorable safety profile. CAPE and its derivatives could potentially function through many molecular mechanisms involving tyrosine kinases and anti-angiogenesis, and have also shown some ability to protect organ function. Therefore, in the clinical setting, these agents might be considered as a therapeutic choice for patients who have contraindication or substantial risks preventing the use of tyrosine kinase inhibitors and antiangiogenic drugs. The present findings warrant further study of CAPE 1ʹ for HCC treatment.

## Data Availability

The data presented in this study are available on request from the corresponding authors.

## Abbreviations

CAPE: *Caffeic acid phenethyl ester*
HCC: Hepatocellular carcinoma
PI: Propidium iodide
IC50: 50% Inhibitory concentration
LD50: Median lethal dose
